# Differential Adaptive Response of Growing Bones From Two Female Inbred Mouse Strains to Voluntary Cage‐Wheel Running

**DOI:** 10.1002/jbm4.10032

**Published:** 2018-02-12

**Authors:** Stephen H. Schlecht, Melissa A. Ramcharan, Yueqin Yang, Lauren M Smith, Erin MR Bigelow, Bonnie T Nolan, Drew E Moss, Maureen J Devlin, Karl J Jepsen

**Affiliations:** ^1^ Department of Orthopaedic Surgery University of Michigan Ann Arbor MI USA; ^2^ US Food and Drug Administration Silver Spring MD USA; ^3^ Wuhan Sports University Wuhan China; ^4^ School of Public Health University of Michigan Ann Arbor MI USA; ^5^ Department of Anthropology University of Michigan Ann Arbor MI USA

**Keywords:** VOLUNTARY CAGE‐WHEEL RUNNING, BONE PHENOTYPE, BONE FUNCTION, MECHANICAL LOADING, A/J AND C57BL6/J MICE

## Abstract

The phenotypic response of bones differing in morphological, compositional, and mechanical traits to an increase in loading during growth is not well understood. We tested whether bones of two inbred mouse strains that assemble differing sets of traits to achieve mechanical homeostasis at adulthood would show divergent responses to voluntary cage‐wheel running. Female A/J and C57BL6/J (B6) 4‐week‐old mice were provided unrestricted access to a standard cage‐wheel for 4 weeks. A/J mice have narrow and highly mineralized femora and B6 mice have wide and less mineralized femora. Both strains averaged 2 to 9.5 km of running per day, with the average‐distance run between strains not significantly different (*p *= 0.133). Exercised A/J femora showed an anabolic response to exercise with the diaphyses showing a 2.8% greater total area (Tt.Ar, *p* = 0.06) and 4.7% greater cortical area (Ct.Ar, *p* = 0.012) compared to controls. In contrast, exercised B6 femora showed a 6.2% (*p* < 0.001) decrease in Tt.Ar (*p* < 0.001) and a 6.7% decrease in Ct.Ar (*p *= 0.133) compared to controls, with the femora showing significant marrow infilling (*p *= 0.002). These divergent morphological responses to exercise, which did not depend on the daily distance run, translated to a 7.9% (*p *= 0.001) higher maximum load (ML) for exercised A/J femora but no change in ML for exercised B6 femora compared to controls. A consistent response was observed for the humeri but not the vertebral bodies. This differential outcome to exercise has not been previously observed in isolated loading or forced treadmill running regimes. Our findings suggest there are critical factors involved in the metabolic response to exercise during growth that require further consideration to understand how genotype, exercise, bone morphology, and whole‐bone strength interact during growth. © 2018 The Authors. *JBMR Plus* is published by Wiley Periodicals, Inc. on behalf of the American Society for Bone and Mineral Research.

## Introduction

Mechanical loads applied to the skeleton during growth are generally considered to promote an anabolic response that results in greater bone mass accumulation.^1^, ^2^, ^3^, ^4^ This adaptive response varies among inbred mouse strains, indicating that it is genetically regulated.^5^, ^6^, ^7^, ^8^ Prior work has reported differential responses of long bones from inbred mouse strains to functionally isolated loading,^5^ forced treadmill running,^9^ jumping,^10^ and unloading.^11^ However, it is less understood how different strains of mice respond to voluntary running. Recently, we reported that A/J and C57BL/6J (B6) mouse femora, which assemble different sets of traits to achieve a similar mechanical homeostatic state at adulthood, show differential regulation of molecular pathways integral to the establishment of external bone size and tissue mineralization during growth.^12^ Adult A/J femoral diaphyses have a narrow external cortex that is thick and highly mineralized (ie, higher ash content), whereas B6 femoral diaphyses have a wider external cortex that is thin and less mineralized. Functionally these morphological and compositional differences translate into A/J femora having similar whole‐bone stiffness and strength compared to B6 femora, but at the expense of having lower postyield deflection and thus more brittle bones. These different mechanisms of functional homeostasis are potentially attributable to growing A/J femora having an inhibited canonical *Wnt* pathway (an important inducer of osteoblastic differentiation) and an induced acidic serine aspartate‐rich *Mepe*‐associated motif (ASARM) bone‐renal pathway (an important inducer of tissue mineralization), relative to B6. Both of these pathways show a greater than twofold functional enrichment between strains, with the majority of *Wnt* antagonist (eg, *Sost*, *Dkks*, *Sfrps*) and ASARM bone‐renal agonist (eg, *Mepe*, *Phex*, *Dmp1*) genes having significantly greater expression levels in A/J femora relative to B6 femora.^12^ Given these gene expression differences, we tested the hypothesis that A/J and B6 femora would show a differential phenotypic response to an increase in physiological loading (ie, voluntary cage‐wheel running) beginning at 4 weeks of age, defined as differences in bone morphology, composition, and mechanical properties between exercised and non‐exercised test groups. Additionally, we tested the hypothesis that given the baseline femoral diaphyseal shape differences between A/J (narrow) and B6 (wide) mice, A/J mice would exhibit greater periosteal expansion in response to exercise compared to their B6 counterparts. Third, we tested whether there was a consistent differential response to voluntary cage‐wheel running across skeletal sites (ie, humeri, vertebrae). Finally, because the amount of voluntary cage‐wheel running is expected to vary among exercised mice, we tested for associations between distance run and each of the morphological and mechanical properties to identify parameters that may be sensitive to the amount of cage‐wheel activity during growth. Though this study does not specifically test for in situ tissue‐level strain differences in the femora of growing A/J and B6 mice, we anticipate that mechanical strain levels will be similar between the two running mouse strains because they both have similar body mass and long bones of near equivalent stiffness.^13^ Thus, any differential phenotypic response to voluntary cage‐wheel running should be more applicable to either a difference in the number of loading cycles (ie, wheel revolutions) sustained, or may reflect a difference in the normal strain distribution pattern between these two mouse strains.

## Materials and Methods

The beginning age and duration of the study was designed to coincide with the prepubertal period that we have previously shown to be when A/J and B6 have the greatest divergence in terms of femoral diaphyseal phenotype and gene expression profiles.^12^

Twenty female A/J and 20 female B6 inbred mice were purchased from the Jackson Laboratory (Bar Harbor, ME, USA) at 3 weeks of age, and allowed 1 week to acclimate before the start of the study. For each strain, mice were placed in a control (*n* = 10) or exercise group (*n* = 10), taking care to have groups with similar body weight distributions, and individually housed for the duration of the study. At the completion of the study an additional cohort of female A/J and B6 female mice (*n* = 5/group/strain) were purchased to repeat the experiment and confirm our initial findings. The outcome of both studies was consistent; therefore, we report the findings for 30 mice per strain (*n* = 15/group/strain). All mice were provided water and fed a standard rodent diet (D12450B; Research Diets, New Brunswick, NJ, USA) *ad libitum*. Mice were kept on a 12‐hour light/dark cycle, and provided a nestlet for cage enrichment. A/J and B6 mice assigned to the exercise group (*n* = 15/strain) were individually housed and had free access (24 hours/day) to a stainless steel cage‐wheel (115 mm outer diameter; Mini‐Mitter Co., Inc., Murrysville, PA, USA) for 4 weeks. Wheel revolutions were monitored daily and distance run was calculated as the number of revolutions × the outer circumference of the wheel × π. Control mice were also individually housed and allowed normal cage activity during the study. Body weight (BW) was recorded three times per week and food weight (FW) was recorded one time per week throughout the course of the experiment. Mice were euthanized at 8 weeks of age, and the left and right quadriceps muscle complex were harvested and weighed. The left and right femora and humeri (*n* = 15/group/strain), along with the L_2_ vertebrae (*n* = 10/group/strain) were harvested, cleaned of soft tissue, and stored in 1× phosphate‐buffered saline (PBS) solution at −40°C. The Institutional Animal Care and Use Committee (IACUC) of the University of Michigan approved all handling and treatment of mice.

### Morphological and compositional traits

Maximum bone length (Le) was measured using a digital micrometer caliper (0.01 mm resolution) for all femora and humeri. Femora, humeri, and vertebrae were imaged using nano–computed tomography (nanoCT) (nanotom‐s; phoenix|x‐ray; GE Sensing and Inspection Technologies, GmbH, Wunstorf, Germany) while submerged in distilled water. The same imaging parameters were used for all bones (tungsten target, 2000 ms timing, 3 averages, 1 skip, 85 kV, and 220 μA tube settings). Image volumes were reconstructed at an 8‐μm voxel size using datos|x reconstruction software (phoenix|x‐ray, GE Sensing and Inspection Technologies, GmbH, Wunstorf, Germany). Gray values were converted to Hounsfield units using a calibration phantom containing air, water, and an hydroxyapatite mimicker (1.69 mg/mL; Gammex, Middleton, WI, USA) as described.^14^

Image analysis was conducted using Microview Advanced Bone Analysis software (v. 2.2; GE Healthcare, Piscataway, NJ, USA). The cortical region of interest (ROI) examined for the femoral and humeral images was 2 mm in length along the midshaft of the diaphysis. For the femur the ROI began immediately distal to the third trochanter and for the humerus the ROI began just distal to the deltoid tuberosity. Delineated ROIs were thresholded on a per sample basis in accordance with Otsu's^15^ method. For both the femoral and humeral midshaft ROIs, total area (Tt.Ar), cortical area (Ct.Ar), marrow area (Ma.Ar), and cortical tissue mineral density (Ct.TMD) were measured for each cross‐section and then averaged across the ROI.

Analysis of the L_2_ vertebrae images involved manually removing the posterior elements to isolate a perimeter of the vertebral body. The vertebral body cortex was then manually segmented from the trabecular bone. The length of the ROI was 60% of the total length of the vertebral body and did not include the cranial or caudal growth plates. Volumes were thresholded in accordance with Otsu's method.^15^ Cross‐sectional traits of the cortical shell included Tt.Ar, Ct.Ar, and Ct.TMD. Scans were also analyzed for trabecular microarchitectural traits within the centrum of the vertebral body along the same length of the cortical analysis. Trabecular traits measured included bone volume fraction (BV/TV), trabecular thickness (Tb.Th), trabecular number (Tb.N), the degree of anisotropy (DA) in the cranial‐caudal direction, and trabecular tissue mineral density (Tb.TMD). DA measured within the secondary spongiosa was estimated using the mean intercept length (MIL) method.^16^, ^17^ The value of DA ranged from 1 (isotropic) to infinity (anisotropic). The same trabecular traits were also measured for the distal metaphysis of the femur. The ROI for the distal femur was located above the intercondyloid fossa, did not include the growth plate, and had a length that was 10% of the total length of the bone.

### Mechanical testing

Whole‐bone mechanical properties were measured for the left femora, left humeri, and L_2_ vertebral bodies of each mouse. All testing was performed using a servohydraulic materials testing system (MTS 858; MiniBionix, Eden Praire, MN, USA) at a displacement rate of 0.05 mm/s. Femora were loaded to failure with the anterior surface in tension and using a custom four‐point bending fixture with the upper supports 2.20 mm apart and the lower supports 6.35 mm apart. Humeri were loaded to failure with the lateral surface in tension using the same four‐point loading fixture and support distances. L_2_ vertebral bodies were loaded to failure in the cranial‐caudal direction using a custom compression fixture. For each femoral, humeral, and vertebral sample, stiffness (S), maximum load (ML), postyield deflection (PYD), and work‐to‐fracture (Work) were calculated from the load‐deflection curves.^18^ In the vertebral compression tests failure occurred in two phases, a structural phase followed by a compaction phase. Failure of the vertebrae was defined by the initial drop in load, which is easily identified and signifies a loss in structural support within the cortical and trabecular architecture. This measure provided a consistent way to quantify failure across test samples and would be considered a physiologically relevant failure event.

### Ash content

Ash content was quantified for the left femora following mechanical testing. Briefly, femoral fragments were cleaned of extraneous soft tissue and bone marrow using a stereomicroscope (S6e; Leica Microsystems Inc., Buffalo Grove, IL, USA). Samples were then hydrated, dried, and ash weights were measured as described.^18^ Ash content was calculated as the percentage of ash weight relative to the hydrated weight.

### Statistical analysis

The number of mice included in the study was statistically powered for analyses of the femora. However, we expanded our analyses to the humeri and vertebrae to test whether the exercise effect observed for the femur was consistent across skeletal sites. All statistical analyses were performed using Minitab v16 (State College, PA, USA) and Prism v7 (GraphPad Software, La Jolla, CA, USA). A Shapiro‐Wilk test was used to determine if the data were normally distributed. A general linear model (GLM) analysis of variance (ANOVA) was used to test for a phenotypic differential response to exercise between strains with body weight included as a covariate. Post hoc comparisons of body weight adjusted traits between controlled and exercised mice within strains were analyzed using a *t* test with significance taken at *p* ≤ 0.05. Finally, a linear regression analysis was conducted for each inbred strain separately using body weight–adjusted traits to identify morphological, compositional, or mechanical properties that show a significant association with the total distance run over the 4‐week study.

## Results

### Differences in body weight and distance run

**Figure 1:**
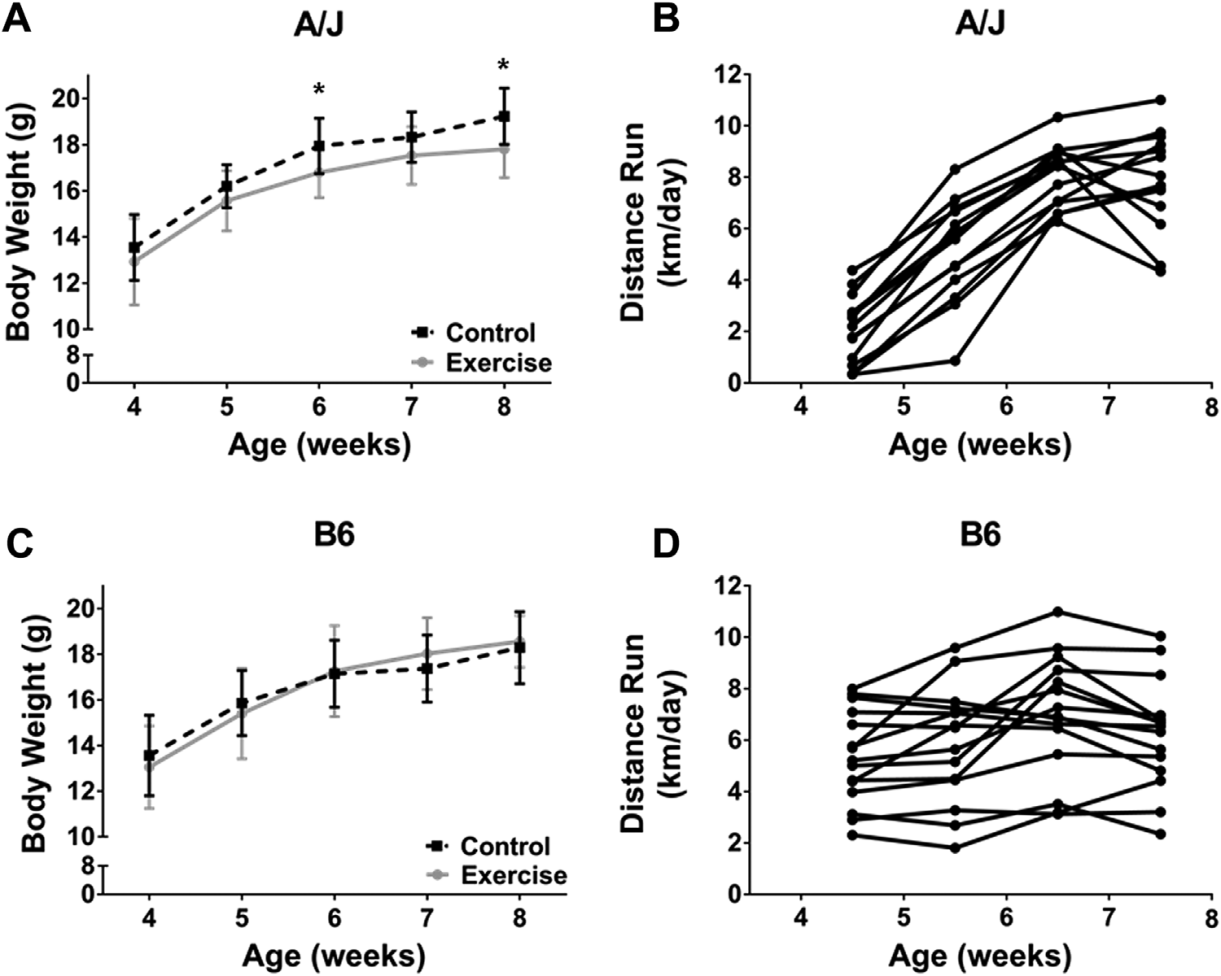
Line plots showing the changes in body weight and distance run for A/J and B6 inbred mouse strains during 4 weeks of voluntary cage‐wheel running beginning at 4 weeks of age. (*A*) Means and standard deviations of A/J control and exercise body weight; (*B*) weekly average of distance run of each A/J‐exercise mouse; (*C*) means and standard deviations of B6 control and exercise body weight; and (*D*) weekly average of distance run of each B6‐exercise mouse. *Significant at the *p* < 0.05 alpha level.

Mean body weights measured at 4 weeks of age were not significantly different between control and exercise groups for both A/J and B6 mouse strains (Fig. 1). Following 4 weeks of voluntary cage‐wheel running, A/J‐exercise mice (8 weeks of age) had significantly lower body weights (17.8 ± 1.2 g; *p* = 0.003) compared to their controls (19.2 ± 1.2 g) (Fig. 1 *A*), whereas B6 mice showed no statistical differences in body weight between exercise (18.6 ± 1.1 g) and control mice (18.3 ± 1.6 g) (Fig. 1 *B*) over the 4‐week time period. As expected, average food intake during the study was significantly higher in the exercise groups of both strains, with A/J‐exercise mice consuming 21% more (*p* < 0.001) and B6‐exercise mice consuming 14% more (*p* < 0.001) food compared to their respective controls.

The average daily distance run on cage wheels over the course of the 4 weeks was not different between A/J‐exercise (7.1 ± 1.4 km/day) and B6‐exercise (6.3 ± 2.1 km/day) mice (*p* = 0.133). However, the running patterns differed between strains. The daily distance run at 4 weeks of age correlated significantly with the distance run at 8 weeks of age for B6‐exercise (*R* 2 = 0.40, *p* = 0.011) but not A/J‐exercise (*R* ^2^ = 0.0006, *p* = 0.933) mice. A/J mice took about 1 week to acclimate to the cage wheel, whereas B6 mice used the cage wheel immediately (Fig. 1 *C*, *D*). Moreover, the body weight at 4 weeks of age was positively correlated with the average daily distance run throughout the 4‐week study by B6 mice (*R* ^2^ = 0.73, *p* < 0.001), but not A/J mice (*p* = 0.196). Thus, B6 mice that were larger at the beginning of the study ran more over the course of 4 weeks.

### Differences in femoral cortices with exercise

**Figure 2:**
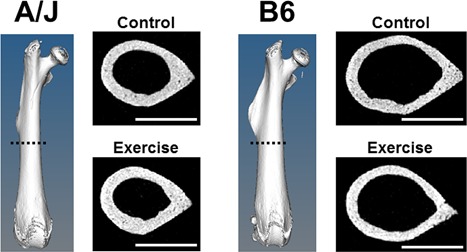
Representative nanoCT images of control and exercise femoral diaphyseal midshafts of A/J and B6 mice at 8 μm voxel size.

**Figure 3:**
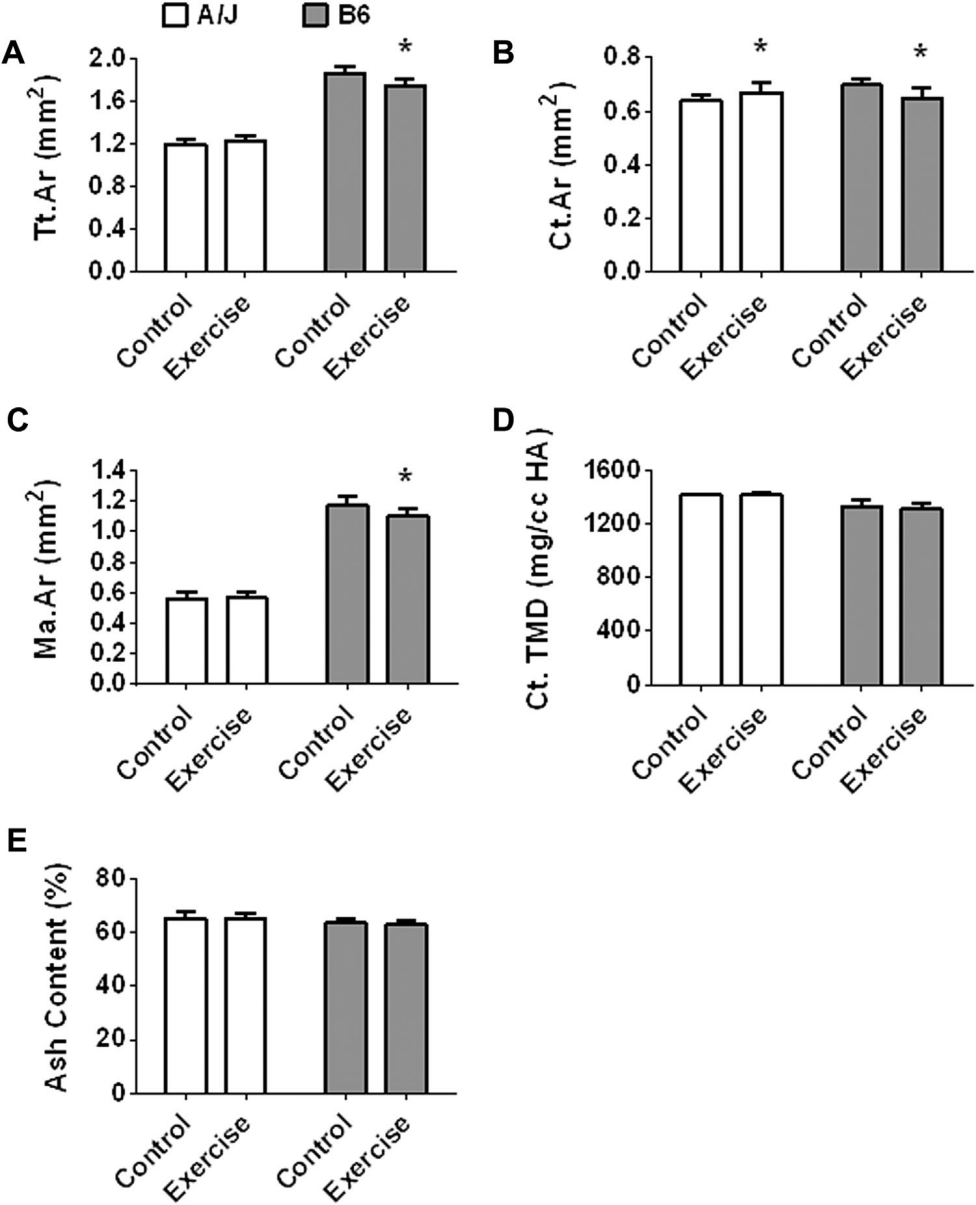
Bar charts showing means and standard deviations of femoral bone morphology and compositional traits of control and exercise A/J and B6 mice after adjusting for body weight. (*A*) Tt.Ar; (*B*) Ct.Ar; (*C*) Ma.Ar; (*D*) Ct.TMD; and (*E*) ash content. Tt.Ar and Ct.Ar were significantly associated (*p* < 0.01 and *p* < 0.001, respectively) with body weight in all A.J and B6 mice. Ma.Ar was significantly associated (*p* < 0.05) with body weight among all B6 mice. Ct.TMD was only significantly associated (*p* = 0.03) with body weight among AJ‐control mice. *Significant at the *p* < 0.05 alpha level.

**Figure 4:**
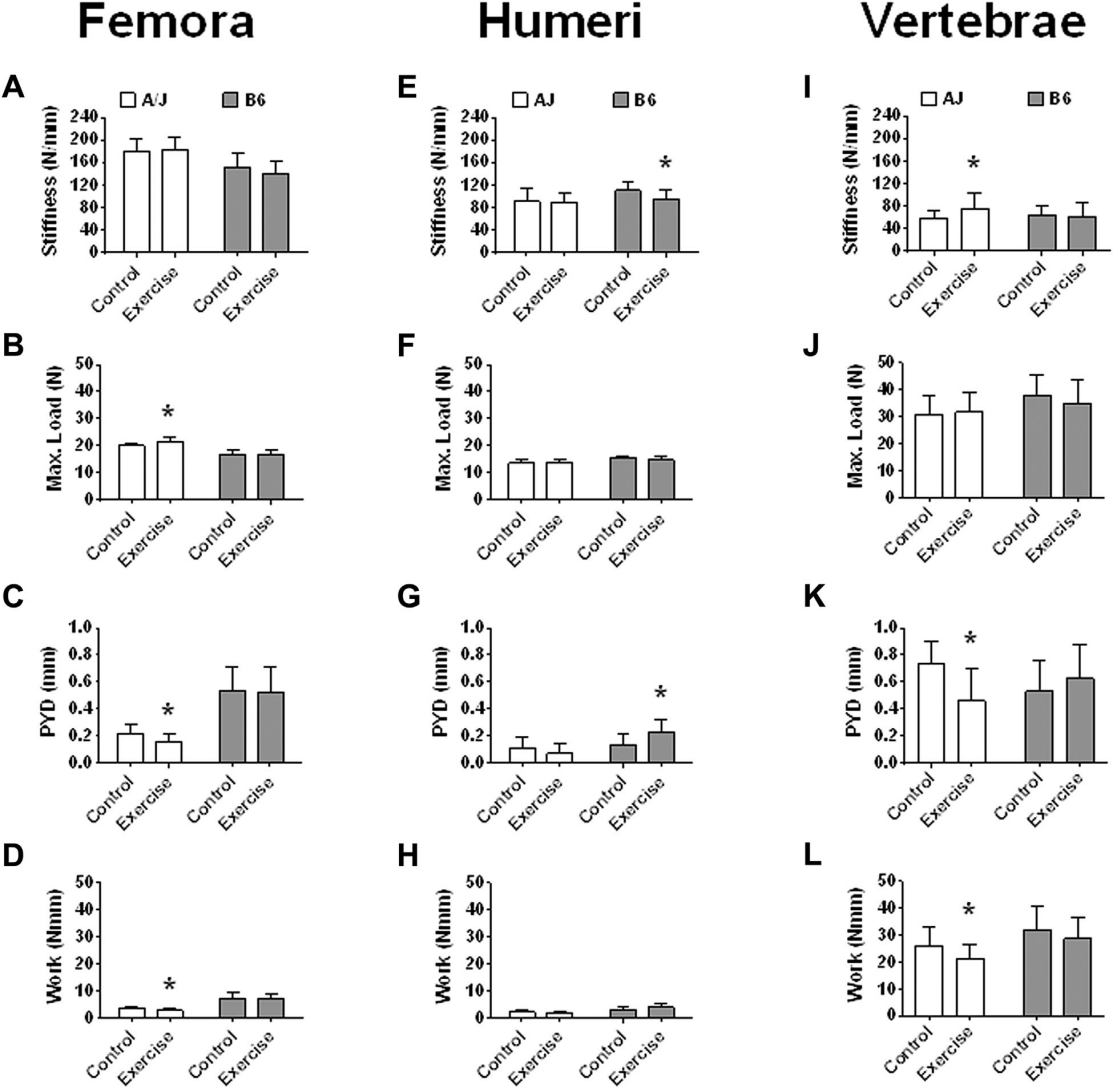
Bar charts showing means and standard deviations of femoral and humeral mechanical properties of control and exercise A/J and B6 mice after adjusting for BW. (*A*) femoral S; (*B*) femoral ML; (*C*) femoral PYD; (*D*) femoral Work; (*E*) humeral S; (*F*) humeral ML; (*G*) humeral PYD; (*H*) humeral Work; (*I*) vertebral S; (*J*) vertebral ML; (*K*) vertebral PYD; and (*L*) vertebral Work. Femoral S was significantly associated (*p* = 0.02) with BW among A/J‐control mice. Femoral ML was significantly associated (*p* < 0.01) with BW among B6‐control mice and all A/J mice. Femoral PYD was significantly associated (*p* < 0.05) with BW among all A/J mice. Humeral S was significantly associated (*p* = 0.02) with BW among B6‐control mice. Humeral maximum load was significantly associated (*p* < 0.05) with BW among all B6 mice. *Significant at the *p* < 0.05 alpha level. BW = body weight; S = stiffness; ML = maximum load; PYD = postyield deflection; Work = work to fracture.

A/J‐control mice demonstrated a narrower femoral diaphysis compared to B6 control mice at 8 weeks of age (*p* < 0.001) (Fig. 2). Comparing the control and exercise mice of each strain there were no statistically significant differences in femoral length (A/J: *p* = 0.083; B6: *p* = 0.361) or quadriceps muscle mass (A/J: *p* = 0.077; B6: *p* = 0.659) after adjusting for body weight. Femoral diaphyses of A/J‐exercise mice showed a 2.8% greater Tt.Ar (*p* = 0.060), a 4.7% greater Ct.Ar (*p* = 0.012), but no change in Ma.Ar (*p* = 0.807) compared to controls (Fig. 3 *A*–*C*). In contrast, femoral diaphyses of B6‐exercise mice showed a 6.2% lesser Tt.Ar (*p* < 0.001), a 6.7% lesser Ct.Ar (*p* < 0.001), and a 5.9% lesser Ma.Ar (*p* = 0.002) compared to controls (Fig. 3 *A*–*C*). Neither strain showed a statistically significant difference in femoral Ct.TMD (A/J: *p* = 0.590; B6: *p* = 0.265) (Fig. 3 *D*) or ash content (A/J: *p* = 0.570; B6: *p* = 0.191) (Fig. 3 *E*) between exercised and control groups. In terms of whole‐bone mechanical properties, A/J‐exercise femora showed no difference in S (*p* = 0.666), a 7.9% greater ML (*p* = 0.001), a 29.3% lesser PYD (*p* = 0.011), and a 19% lesser Work (*p* = 0.022) compared to controls (Fig. 4 *A*–*D*). However, B6‐exercise femora showed no statistically different mechanical properties compared to their controls.

### Differences in humeral cortices with exercise

Similar to the femoral diaphyses, A/J control mice had a narrower humeral diaphysis compared to B6‐control mice at 8‐weeks of age (*p* < 0.001) (Table 1). Additionally, there was no significant difference in humeral length (A/J: *p* = 0.641; B6: *p* = 0.381) after adjusting for body weight between the exercise and control groups of both strains. Similar to the effects of running observed in the femora, Ct.Ar of the humeral midshaft was 3.5% greater in A/J‐exercise mice compared to controls (*p* = 0.02), and 4.2% lower in B6‐exercise mice compared to controls (*p* = 0.02). The effects of running on the humeri was also similar to that observed in the femora with a 3.3% greater Tt.Ar in A/J‐exercise mice (*p* = 0.145) and a 3.9% lesser Tt.Ar in B6‐exercise mice (*p* = 0.096), though neither was significant. Functionally, A/J‐exercise humeri showed no statistical differences in whole bone mechanical properties compared to the controls (Fig. 4 *E*–*H*). However, B6‐exercise humeri showed a 14.1% lesser S (*p* = 0.047), a 4.9% lesser ML (*p* = 0.098), a 65.1% greater PYD (*p* = 0.029), and a 30.5% greater Work (*p* = 0.075) compared to the controls.

**Table 1:** Means and Standard Deviations of the Morphological and Compositional Parameters of the Humeral Midshafts, L2 Vertebral Bodies, and Distal Femora of 8‐Week‐Old Control and Exercised Female A/J and B6 Mice

	A/J		B6	
	Control	Exercise	Control	Exercise
Humeral midshaft				
Tt.Ar (mm^2^)	0.65 ± 0.04	0.68 ± 0.01^a^	0.82 ± 0.03^a^	0.80 ± 0.03^a^
Ct.Ar (mm^2^)	**0.415 ± 0.015** ^a^	0.432 ± 0.009^a^	**0.446 ± 0.011** ^a^	0.430 ± 0.026^a^
Ma.Ar (mm^2^)	0.24 ± 0.03	0.24 ± 0.01^a^	0.38 ± 0.02^a^	0.37 ± 0.02^a^
Ct.TMD (mg/mL HA)	1345 ± 19^a^	1401 ± 78^a^	1328 ± 17	1321 ± 27
L_2_ vertebral body				
Tt.Ar (mm^2^)	1.23 ± 0.04	1.25 ± 0.05^a^	1.39 ± 0.04^a^	1.38 ± 0.07^a^
Ct.Ar (mm^2^)	0.304 ± 0.013	0.308 ± 0.014^a^	0.304 ± 0.015^a^	0.297 ± 0.021
Ma.Ar (mm^2^)	0.92 ± 0.03	0.94 ± 0.04^a^	1.09 ± 0.04^a^	1.08 ± 0.05^a^
Ct.TMD (mg/mL HA)	1101 ± 33	1110 ± 42	1049 ± 43^a^	1031 ± 39
BV/TV (%)	20 ± 1	20 ± 2	25 ± 2	24 ± 2
Tb.Th (mm)	**0.032 ± 0.001**	**0.033 ± 0.001**	**0.033 ± 0.001**	**0.032 ± 0.001** ^a^
Tb.N (mm^–1^)	6.16 ± 0.29	6.22 ± 0.30	7.45 ± 0.38	7.37 ± 0.44
DA	2.09 ± 0.11	2.13 ± 0.10^a^	2.12 ± 0.20	2.07 ± 0.15
Tb.TMD (mg/mL HA)	898 ± 32	914 ± 29	872 ± 31^a^	862 ± 36
Distal femur				
BV/TV (%)	**21 ± 2**	**23 ± 1**	17 ± 2	16 ± 2^a^
Tb.Th (mm)	**0.034 ± 0.001**	**0.035 ± 0.001**	**0.030 ± 0.001**	**0.029 ± 0.001**
Tb.N (mm^–1^)	6.36 ± 0.30	6.43 ± 0.21	5.54 ± 0.40	5.36 ± 0.68^a^
DA	1.45 ± 0.06	1.49 ± 0.08	1.54 ± 0.07	1.52 ± 0.09
Tb.TMD (mg/mL HA)	**995 ± 18** ^a^	**1026 ± 11** ^a^	**870 ± 57**	**842 ± 56**

All values given are adjusted for body weight. Values denoted in bold indicate significant differences (*p* < 0.05) between the control and exercised mice within each mouse strain.

HA = hydroxyapatite.

^a^ Denotes traits that are significantly (*p* < 0.05) associated with body weight.

Differences in trabecular architecture in distal femur and vertebral bodies with exercise A/J‐exercise mice showed significantly more trabecular BV/TV (*p* = 0.022), Tb.Th (*p* < 0.001), and Tb.TMD (*p* < 0.001) in the distal femur and Tb.Th (*p* = 0.041) in the vertebral body compared to controls (Table 1). B6‐exercise mice showed significantly less Tb.Th for the vertebral body (*p* = 0.04) and distal femur (*p* = 0.016) compared to controls. Functionally, only A/J‐exercise mice showed significant differences in vertebral mechanical properties compared to the controls (Fig. 4 *I*–*L*). PYD was 35% lower (*p* = 0.002) and Work was 21% lower (*p* = 0.026) in the L_2_ vertebrae of the exercise group.

### Effects of distance run on bone traits at 8 weeks of age

**Figure 5:**
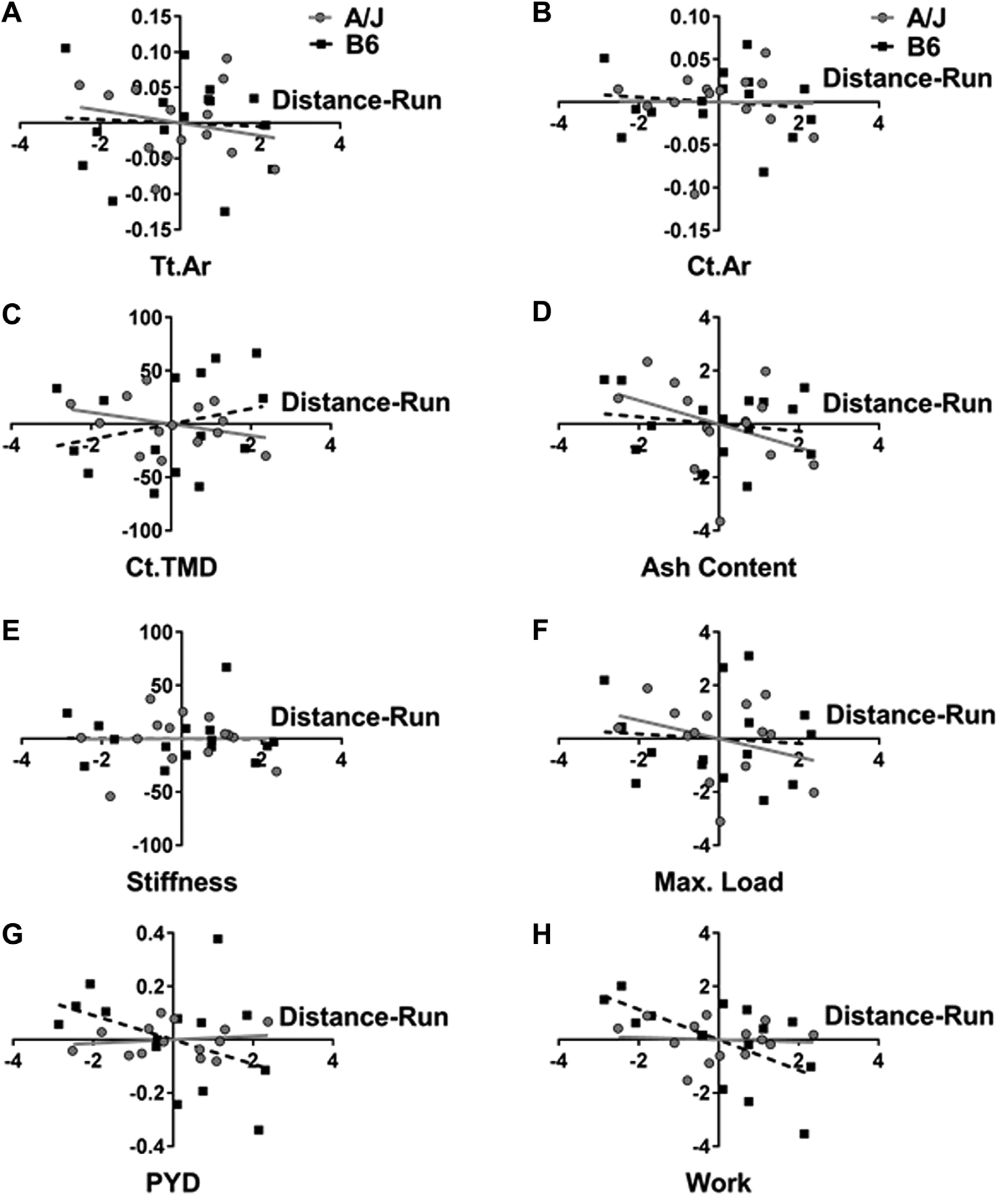
Linear regressions between femoral morphological, compositional, and mechanical properties and the total distance run over 4 weeks after adjusting for body weight. (*A*) Tt.Ar; (*B*) Ct.Ar; (*C*) Ct.TMD; (*D*) ash content; (*E*) stiffness; (*F*) maximum load; (*G*) PYD; and (*H*) Work. PYD = postyield deflection; Work = work to fracture.

Comparisons between body weight adjusted bone traits of all skeletal sites and the average distance run over 4 weeks by each mouse identified only a few bone traits that were associated with the amount of running each mouse performed (Table 2, Fig. 5 *A–G*). A significant negative correlation was found between Work and distance run for B6‐exercise femora (*R* ^2^ = 0.36, *p* = 0.017), but not A/J‐exercise femora (Fig. 5 *H*). A significant positive correlation with distance run was also found for Ct.TMD (*R* ^2^ = 0.30, *p* = 0.036) and whole‐bone stiffness (*R* ^2^ = 0.53, *p* = 0.017) for B6‐exercise humeri.

**Table 2:** Outcomes of Partial Regression Analysis for Cortical and Trabecular Bone Traits and Mechanical Properties Relative to the Distance Run

		A/J			B6		
	Element	R^2^	p	Slope	R^2^	p	Slope
Cortical bone							
Tt.Ar (mm^2^)	Femora	0.037	0.492	–	0.004	0.832	–
	Humeri	0.025	0.586	–	0.067	0.353	+
	Vertebrae	0.041	0.485	–	0.093	0.269	+
Ct.Ar (mm^2^)	Femora	0.004	0.813	+	0.015	0.660	–
	Humeri	<0.0001	0.950	–	0.103	0.244	+
	Vertebrae	0.036	0.515	–	0.011	0.712	+
Ct.TMD (mg/mL HA)	Femora	0.024	0.581	–	0.069	0.345	+
	Humeri	0.018	0.648	+	**0.295**	**0.036**	+
	Vertebrae	0.035	0.522	–	0.028	0.554	+
Trabecular bone							
BV/TV (%)	Vertebrae	0.005	0.814	+	0.067	0.352	–
	Distal femora	<0.0001	0.976	–	0.134	0.180	–
Tb.Th (mm)	Vertebrae	0.025	0.590	+	0.105	0.293	+
	Distal femora	0.003	0.856	+	0.054	0.405	+
Tb.N (mm^–1^)	Vertebrae	<0.001	0.985	–	0.240	0.064	–
	Distal femora	0.004	0.831	+	0.176	0.119	–
DA	Vertebrae	0.048	0.452	+	0.016	0.650	–
	Distal femora	0.006	0.783	–	0.207	0.089	–
Tb.TMD (mg/mL HA)	Vertebrae	0.019	0.641	–	0.022	0.595	+
	Distal femora	0.001	0.934	+	0.158	0.142	+
Bone mechanics							
Stiffness (N/mm)	Femora	0.011	0.716	+	<0.0001	0.972	–
	Humeri	0.219	0.173	–	**0.530**	**0.017**	+
	Vertebrae	<0.0001	0.992	+	0.109	0.230	–
Maximum load	Femora	0.222	0.076	–	0.008	0.756	–
	Humeri	0.076	0.440	+	0.238	0.153	+
	Vertebrae	0.003	0.862	–	**0.346**	**0.021**	–
PYD	Femora	0.023	0.588	–	0.159	0.141	–
	Humeri	0.002	0.915	–	0.179	0.224	–
	Vertebrae	0.014	0.692	+	0.161	0.138	+
Work	Femora	0.020	0.618	–	**0.364**	**0.017**	–
	Humeri	0.245	0.146	+	0.002	0.915	+
	Vertebrae	0.003	0.857	+	**0.283**	**0.041**	–

All values given are adjusted for body weight. Positive (+) and negative (–) slope directions are indicated. Values denoted in bold indicate significant (*p* < 0.05) associations.

HA = hydroxyapatite.

## Discussion

Our data support the hypothesis that A/J and B6 femora will demonstrate a differential phenotypic response to increased physiological activity beginning at 4 weeks of age. Voluntary cage‐wheel running was used to avoid a stress response that may be associated with forced treadmill running.^19^ Once both strains were adjusted to the wheel (∼4 days), A/J and B6 mice voluntarily ran between 2 and 9.5 km per day over the 4 week study. The majority of this running occurred at night. The lowest distance run after the first 4 days of the study was 2 km/day for A/J and 5 km/day for B6, which is ∼85% to 95% further than the typical distance run after 30 min on a forced treadmill at a rate of 12 m/min.^20^, ^21^, ^22^ Despite mice being genetically homogenous within each strain, there was tremendous variation in the number of revolutions each mouse chose to run each day. Further, the number of revolutions each mouse ran each day tended to be consistent across the study, thereby creating low‐distance and high‐distance runners in each strain.

Exercise led to an anabolic response in A/J femoral diaphyses with more Ct.Ar compared to controls. The change in Tt.Ar but not Ma.Ar between A/J‐exercise and A/J‐control indicated that the greater mass resulted from a larger outer bone size or periosteal expansion rather than marrow infilling. The larger cortical volume resulted in greater whole‐bone strength. However, A/J‐exercise mice showed significantly less postyield displacement and work‐to‐fracture compared to controls, indicating that exercise resulted in a more brittle phenotype. Surprisingly, B6‐exercise mice showed a narrower femoral diaphysis combined with lower cortical area relative to their controls. This reduction among morphologic traits did not adversely affect the mechanical function of B6 femora, as there was no difference among controlled and exercised mice in terms of mechanical properties. Taken together, this data demonstrated that access to a cage wheel during growth was associated with a divergent morphological response in A/J and B6 femora, with A/J mice showing an anabolic‐type response with greater periosteal expansion and mass accumulation and B6 mice showing suppressed periosteal expansion and mass accumulation.

Though this study was only statistically powered for the femur, the divergent morphological response observed for the femora was generally consistent for the humeri but not the vertebrae. Like the femora, the humeri showed greater Ct.Ar for A/J‐exercise mice but lesser Ct.Ar for B6‐exercise mice compared to controls. These changes in mass accumulation resulted from small changes in Tt.Ar but not Ma.Ar, suggesting periosteal expansion and not marrow infilling was affected by the cage‐wheel exercise during growth. The L_2_ vertebral bodies of A/J‐exercise and B6‐exercise mice showed no differences in any cortical or whole‐bone traits compared to their controls, and thus voluntary cage‐wheel running did not appear to alter the vertebral body.

The thinner cortex in B6‐exercise femora and humeri compared to their controls, which occurred through less periosteal and endosteal bone deposition, was unexpected. The smaller outer bone size in B6‐exercise femora was opposite to expectations that exercise should be associated with an anabolic response,^23^ which motivated the replication of the study with five additional mice per group and strain. The only other known voluntary cage‐wheel running study reported in growing B6 female mice femora showed more than a doubling of periosteal area following 4 weeks of running.^24^ However, these mice had access to the cage wheel between 7 and 11 weeks of age. Similarly, Styner and colleagues^25^ voluntarily ran 8‐week‐old B6 female mice for 8 weeks and showed a significant increase in tibial Ct.Ar but no difference in Tt.Ar. In a separate cohort, Styner and colleagues^26^ found no significant phenotypic effect on the tibial diaphysis of mature (16 weeks old) female B6 mice following 6 weeks of voluntary cage‐wheel running. Isakkson and colleagues^27^ conducted a long‐term voluntary running study (24 weeks) beginning at 4 weeks of age in B6 males, and found no significant difference in total femoral cross‐sectional area at 8 weeks of age compared to their controls. Whether the differences in age of the B6 mice among these studies impacted the response of the femora and tibias to exercise is unknown. Nonetheless, our outcome and that of Isakkson and colleagues^27^ is surprising considering that studies conducted on tennis players showed that competitive prepubertal training resulted in an anabolic response in the dominant racket arm versus the nondominant contralateral arm.^1^, ^3^ Several other studies that have mechanically perturbed B6 mice and other select inbred strains during growth have shown a significant periosteal anabolic response similar to that of our A/J‐exercise mice but at a much greater magnitude. However, these mice were either selectively bred for high‐volume voluntary running (HSD:ICR strains)^28^ or the study began during puberty (∼8 weeks of age) and the bones were mechanically perturbed using forced running on treadmills,^22^, ^29^ in vivo mechanical loading,^5^, ^30^, ^31^ or shock‐plate–induced jumping.^30^, ^32^ The discrepancies between our findings and those of others suggest that the adaptive response to loading is dependent on the skeletal site loaded, the loading regime used, along with the strain, sex, and age of the mouse.

Our findings in B6‐exercise mice are opposite to what others have shown in this strain using functionally isolated loading models. One rationale for using a functionally isolated loading paradigm is to reduce the number of confounding factors so that direct correlations can be drawn between an applied load and a subsequent adaptive response. However, we suspect that the mechanical response of bone to loading is also moderated by additional factors such as metabolism, body composition, muscularity, onset of puberty, and others.^33^, ^34^, ^35^ The current study did not test for any of these potential confounding variables. However, the voluntary cage‐wheel running model showed that the distance run by each mouse did not have a noticeable effect on external bone size or whole‐bone strength, suggesting that other factors were present across the exercise group. The lack of an effect of distance run on the morphological traits may reflect that relatively few cycles are needed to exceed baseline threshold levels to generate a bone response, as others have shown.^36^ Whether differential responses to the same training regimen are apparent among humans in different long bones within an individual has not been previously considered. Given that the functional adaptation process of A/J and B6 long bones translate very well to human long bones during growth^37^ and aging,^38^ future work should consider testing whether the adaptive response to specific exercise patterns is uniform within human populations.

The differential adaptive response of bone to exercise between A/J and B6 mice may be related to genotypic differences as reported.^12^ A/J mice demonstrate significant inhibition of the *Wnt/β‐catenin* pathway between 4 and 8 weeks of age, whereas B6 mice show induction of this pathway during the same time frame. Though we did not analyze gene expression profiles of our exercised mice in this study, we speculate that exercise altered this pathway, with increased activity levels leading to a more induced *Wnt* pathway in A/J mice and greater induction of this pathway in B6 mice. In contrast to work by others showing a greater mineral:matrix ratio in the tibias of adult B6 mice following 3 weeks of forced treadmill running,^39^ voluntary exercise during growth had no significant effect on cortical mineralization in either strain, as measured through the analysis of Ct.TMD and ash content. Taken in the context of what we previously reported in these mice between the ages of 4 and 8 weeks of age concerning the ASARM bone renal pathway, an important contributor to tissue mineralization, it would appear that the differences in the induction (A/J) and inhibition (B6) of this pathway observed in less active (control) mice was not affected with the increase in activity levels in the runners as the intrinsic differences in ash content between A/J and B6 mice were maintained in the exercise cohorts.

A mouse exercise model using voluntary cage‐wheel running was used to test the adaptive bone response to exercise in a physiologically relevant way and to detect subtle effects in a manner that best mimics daily loads experienced by active juveniles during growth. This mode of exercise stimulus in mice has been found to minimize physiological stressors, is cognizant of mouse nocturnal activity patterns, is more consistent with their endurance exercise capacity, and is amenable to the short bursts (∼150 s) mice prefer to run.^19^ Additionally, mice participating in voluntary exercise have been shown to run at speeds that conform to those achieved via treadmill experiments. Our study contributed to this literature by showing divergent morphological responses of two strains of mice to voluntary cage‐wheel running and that this response did not depend on the distance run. However, there were some limitations in our study worth noting. First, we did not characterize the cellular bone remodeling activity (ie, osteoblasts and osteoblasts) in the femora of mice between the ages of 4 and 8 weeks, which others have shown^40^ may vary between inbred mouse strains of the same age. However, the purpose of our study was to examine whether exercise had a differential effect on bone in mice of established phenotypic and genotypic background differences that mirror the variation within bone trait sets observed during growth^41^, ^42^ and upon adulthood^37^, ^43^ among humans. To convincingly confirm our hypotheses, a similar study is needed using a cohort of children of the same age. Second, A/J mice are behaviorally more timid than B6 showing reduced open field activity,^44^ which in the current study may help explain why A/J mice took at least 1 week of exposure to the cage‐wheel to match the daily revolutions run by their B6 counterparts. Subsequent unpublished running studies in our laboratory have found that delaying B6 running by 1 week results in more similar running patterns between the strains. Though the use of the cage‐wheel was delayed among A/J mice, they ultimately surpassed the daily revolutions logged by B6 mice. Therefore, even though A/J exercise mice began running at slightly older ages, their 4‐week average daily distance run was not significantly different than that of the B6 mice. Third, our analysis of bone traits was conducted at the completion of the study, and did not include baseline measures of the bone traits prior to the introduction of the cage wheels. Therefore, we were unable to compare the developmental changes that occurred within traits to the distance run by the mice. However, as mentioned previously in the Results, there was no significant difference between bone traits of mice that comprised the exercise group of each strain and the number of revolutions they performed, suggesting that the variation in distance run by all mice was within the same activity threshold range.^45^ Nevertheless, to better understand how exercise influences bone traits and to control for other confounding factors, future work should obtain readily available baseline and endpoint measures pertaining to the metabolic profile (eg, serum markers), body composition (eg, DXA fat measures), and muscle strength (eg, grip tests) of each mouse. Additionally, future work should measure how gene expression profiles change at various stages (ie, 1, 2, 3, or 4 weeks) of exercise that can then be compared to the phenotypic bone outcomes. Last, the differential bone response reported herein was only studied in female mice. It is unclear whether males, which have been shown to voluntarily run less on a daily basis,^19^ will show a similar adaptive response to that of females.

In conclusion, our data confirmed the hypothesis that bone morphology and strength of A/J and B6 mice will show a differential response to physiological loading during growth. A novel finding of this study was that voluntary cage‐wheel running elicited an anabolic response in a mouse strain (A/J) that tends to have narrow long‐bones, while it suppressed periosteal expansion in a mouse strain (B6) that tends to have wide long‐bones. This outcome has not been observed in prior studies using isolated loading or forced treadmill running regimes. Thus, our findings suggest there are critical factors involved in the metabolic response to exercise during growth that require consideration to understand how genotype, exercise, bone morphology, and whole‐bone strength interact with one another during growth. Future genomic and metabolic work is needed to identify the potential mechanism responsible for the differential phenotypic bone response to exercise in these two mouse strains.

## Disclosures

All authors state they have no conflicts of interest.
